# Hypoxia and Inflammation: Insights From High-Altitude Physiology

**DOI:** 10.3389/fphys.2021.676782

**Published:** 2021-05-26

**Authors:** Kathy Pham, Keval Parikh, Erica C. Heinrich

**Affiliations:** Division of Biomedical Sciences, School of Medicine, University of California, Riverside, Riverside, CA, United States

**Keywords:** hypoxia, inflammation, high altitude, hypoxia inducible factor, nuclear factor-κB

## Abstract

The key regulators of the transcriptional response to hypoxia and inflammation (hypoxia inducible factor, HIF, and nuclear factor-kappa B, NF-κB, respectively) are evolutionarily conserved and share significant crosstalk. Tissues often experience hypoxia and inflammation concurrently at the site of infection or injury due to fluid retention and immune cell recruitment that ultimately reduces the rate of oxygen delivery to tissues. Inflammation can induce activity of HIF-pathway genes, and hypoxia may modulate inflammatory signaling. While it is clear that these molecular pathways function in concert, the physiological consequences of hypoxia-induced inflammation and how hypoxia modulates inflammatory signaling and immune function are not well established. In this review, we summarize known mechanisms of HIF and NF-κB crosstalk and highlight the physiological consequences that can arise from maladaptive hypoxia-induced inflammation. Finally, we discuss what can be learned about adaptive regulation of inflammation under chronic hypoxia by examining adaptive and maladaptive inflammatory phenotypes observed in human populations at high altitude. We aim to provide insight into the time domains of hypoxia-induced inflammation and highlight the importance of hypoxia-induced inflammatory sensitization in immune function, pathologies, and environmental adaptation.

## Introduction

Inflammation plays a key role in the physiological response to hypoxic stress. Tissues experience hypoxia during injury, infection, hypoperfusion, ischemia, or hypoxemia secondary to sleep apnea, pulmonary disease, anemia, high-altitude exposure, or other causes (Celli et al., 2004; McNicholas, 2009; Brill and Wedzicha, 2014; Hirota, 2015; Couzin-Frankel, 2020; Tobin et al., 2020; Xie et al., 2020). Cellular hypoxia can trigger the expression of several inflammatory mediators which signal tissue damage and initiate survival responses. However, while hypoxia-induced inflammation may serve a protective role by initiating an immune response and promoting tissue healing, it can also contribute to several pathologies, particularly in the context of chronic hypoxia. In this review, we summarize the known crosstalk between the transcriptional responses to hypoxia and inflammation and highlight the physiological consequences that occur as a result of maladaptive hypoxia-induced inflammation. Finally, we review what we know about adaptive regulation of inflammation under chronic hypoxia by investigating inflammatory phenotypes in human populations adapted to high altitude.

## Molecular Mechanisms

### Hypoxia-Inducible Factor Pathway

**Figure 1:**
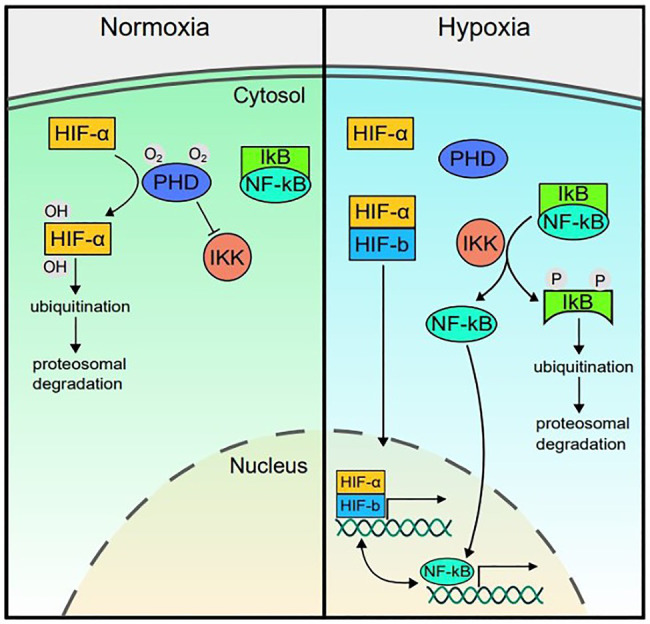
Hypoxia inducible factor (HIF)-nuclear factor (NF)-kappa B (NF-κB) crosstalk. In normoxic conditions, prolyl hydroxylases (PHDs) hydroxylate HIF-α and the IKKβ subunit of the IκB kinase (IKK) complex, marking them for degradation and thereby reducing transcriptional activity of HIF and repressing (but not completely blocking) NF-kB activity. In hypoxia, PHD activity decreases since it utilizes oxygen as a cofactor. Therefore, HIF-α is stabilized and can dimerize with the constitutively active HIF-β subunit. The complex translocates to the nucleus to upregulate expression of genes involved in the hypoxia response. In hypoxia, with reduced PHD activity, the rate of IKK degradation of IκB increases, releasing repression of NF-κB and allowing it to translocate to the nucleus at higher rates and upregulate inflammatory gene expression. PHD, prolyl hydroxylase; HIF, hypoxia-inducible factor; NF-κB, Nuclear factor kappa B; IKK, IκB kinase complex (composed of IKK-α, IKK-β, and IKK-γ subunits); and IκB, nuclear factor of kappa light polypeptide gene enhancer in B-cells inhibitor.

The transcriptional response to hypoxia is controlled by the hypoxia-inducible factor (HIF) signaling cascade (Semenza, 2009). HIF is a heterodimer protein composed of an oxygen-sensitive alpha subunit and constitutively expressed beta subunit (Biddlestone et al., 2015). The three HIF isoforms (HIF-1, HIF-2, and HIF-3) have some overlapping roles but also demonstrate distinct functions in different cell types (Carroll and Ashcroft, 2006; Dengler et al., 2014; Watts and Walmsley, 2019). Under normoxic conditions, HIF-α is hydroxylated by oxygen-dependent prolyl hydroxylases (PHDs 1–3 in humans; Figure 1). Upon hydroxylation, HIF-α is ubiquitinated by the von Hippel-Lindau tumor suppressor protein (pVHL) and degraded by proteasomes. Since PHD requires oxygen as a co-substrate, its activity decreases under hypoxic conditions, allowing HIF-α to dimerize with HIF-β and translocate to the nucleus. The HIF complex can then bind to hypoxia response elements (5'-RCGTG-3') in gene promoters to regulate expression of at least 100 genes to coordinate increased oxygen supply to hypoxic tissue (Kaelin and Ratcliffe, 2008; Semenza, 2009). HIF pathway activity is associated with activation of genes involved in metabolic adaptation, such as phosphoglycerate kinase (*PGK*) and lactate dehydrogenase A (*LDHA*), vascularization *via* vascular endothelial growth factor (*VEGF*), as well as red blood cell production *via* erythropoietin (*EPO*), and several other genes involved in improving oxygen delivery and use efficiency (Dengler et al., 2014; Villafuerte et al., 2014).

### HIF and NF-κB Crosstalk

While the HIF pathway primarily responds to hypoxia, HIF expression is also increased in response to non-hypoxic stimuli, including bacterial lipopolysaccharide (LPS), tumor necrosis factor-α (TNF-α), reactive oxygen species, hepatocyte growth factor, and interleukin (IL)-18 *via* crosstalk with the nuclear factor-κB (NF-κB) pathway (Figueroa et al., 2002; Zhou et al., 2003; Frede et al., 2006; Taylor, 2008). The NF-κB transcription factor is a master regulator of inflammation. NF-κB is kept localized in the cytoplasm by inhibitory IκB proteins (Lawrence, 2009; Oeckinghaus and Ghosh, 2009), which thereby inhibit DNA binding by NF-κB (Beg and Jr, 1993; Mitchell et al., 2016). In response to inflammatory stimuli and microbial products, the IκB kinase (IKK) complex phosphorylates IκB, leading to IκB ubiquitination and proteasomal degradation (Israël, 2010). With the degradation of IκB, NF-κB can translocate to the nucleus and upregulate key downstream inflammatory pathways (Hayden and Ghosh, 2004; Perkins, 2006; Mitchell et al., 2016; Figure 1).

The NF-κB pathway can also upregulate HIF-1α (BelAiba et al., 2007). NF-κB subunits bind to the NF-κB binding element within the HIF-1α gene promoter region and induce HIF-1α mRNA expression (Van Uden et al., 2008). Several studies support this NF-κB-dependent HIF-1α expression. For example, in cell culture models (HEK293 cells and pulmonary artery smooth muscle cells); NF-κB transfection resulted in increased HIF-1α mRNA and protein expression. Additionally, when these cells were co-transfected with a mutated dominant negative IκB (which cannot be phosphorylated by IKK) to reduce NF-κB translocation to the nucleus, HIF-1α mRNA and protein expression decreased (BelAiba et al., 2007; Bonello et al., 2007; Görlach and Bonello, 2008).

In addition to this direct link, the HIF and NF-κB pathways also share common regulators. Like HIF-α, the IKK complex responsible for regulating NF-κB activity is also a target of PHD and therefore its NF-κB regulatory activity is oxygen dependent. In normoxic conditions, PHD hydroxylates IKKβ, therefore repressing NF-κB nuclear translocation and transcriptional activity. When PHD is rendered inactive in hypoxic conditions, the IKK complex can proceed to remove IκB from NF-κB, increasing its rate of translocation to the nucleus and upregulating inflammatory gene expression (Figure 1; Cummins et al., 2006; Taylor, 2008).

## Pathophysiological Consequences

### Hypoxia-Induced Inflammation: Adaptive or Maladaptive?

**Figure 2:**
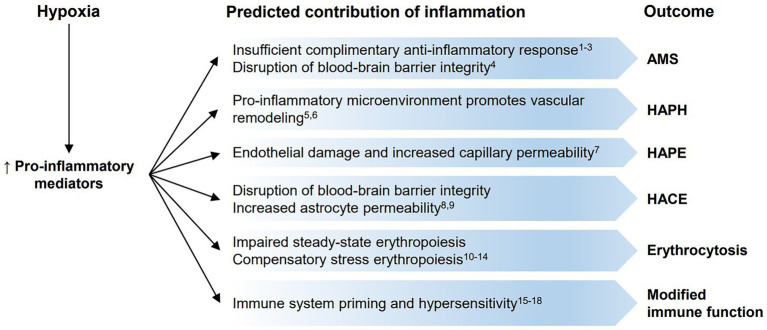
Predicted contributions of inflammation to high-altitude illnesses, erythrocytosis, and immune function. Hypoxic conditions lead to increases in pro-inflammatory mediators, which may play a role in the development of high-altitude illnesses [(Acute Mountain Sickness (AMS), high-altitude pulmonary hypertension (HAPH), high-altitude pulmonary edema (HAPE), and high-altitude cerebral edema (HACE)] and erythrocytosis, or modify immune function. (1) Julian et al. (2011); (2) Liu et al. (2017); (3) Wang et al. (2018); (4) Varatharaj and Galea (2017); (5) Wilkins et al. (2015); (6) Brito et al. (2020); (7) Mishra et al. (2016); (8) Song et al. (2016); (9) Zhou et al. (2017); (10) Jackson et al. (2010); (11) Liao et al. (2018); (12) Bennett et al. (2019); (13) Imagawa et al. (1990); (14) Haase (2013); (15) Kvamme et al. (2013); (16) Baze et al. (2011); (17) Facco et al. (2005); and (18) Feuerecker et al. (2019).

At short timescales, and at the tissue level, inflammatory signaling in response to hypoxia is an adaptive mechanism which evolved to promote cell survival during infection, injury, or oxygen limitation (Walmsley et al., 2014). However, chronic and/or systemic hypoxia can produce maladaptive inflammation which can contribute to disease development. For example, in a clinical context, the crosstalk between hypoxia and inflammation may contribute to several inflammation-mediated metabolic and cardiovascular comorbidities that accompany hypoxia-promoted diseases such as chronic obstructive pulmonary disease or obstructive sleep apnea (Tasali and Ip, 2008; Quercioli et al., 2010; Cavaillès et al., 2013). This can also be investigated in the context of high-altitude exposure, where inflammatory signaling pathways and immune function must respond and adapt to acute, chronic, or lifelong hypoxemia. Here, we will examine our current understanding of how the interaction between hypoxia and inflammation contributes to high-altitude illness and what we might learn about the adaptive regulation of hypoxia-induced inflammation from high-altitude native populations (Figure 2).

### The Role of Inflammation in High-Altitude Illness

High-altitude illnesses are a common risk for sojourners to high altitude (>2,500 m elevation). Within the first week of exposure, sojourners often present with Acute Mountain Sickness (AMS), which is characterized by headache, nausea, fatigue, and gastrointestinal issues (Roach et al., 2018). AMS typically resolves with acclimatization (Gallagher and Hackett, 2004). However, in serious cases, sojourners may develop severe and potentially fatal illnesses, such as high-altitude pulmonary edema (HAPE), high-altitude pulmonary hypertension (HAPH), or high-altitude cerebral edema (HACE; Gallagher and Hackett, 2004; Mehta et al., 2008; Luks et al., 2017). The incidence and severity of HAPH, HAPE and HACE vary depending on multiple factors, such as ascent time, altitude, and timely recognition and treatment (Gallagher and Hackett, 2004). Despite several decades of examining the physiology of AMS, HAPH, HAPE, and HACE, some questions remain regarding the pathophysiology of these conditions and the extent to which inflammation contributes to their onset and progression.

#### Acute Mountain Sickness

AMS development results from a complex network of physiological responses to hypoxia (i.e., inflammation, vasogenic edema, and acidosis) as well as anatomical factors (i.e., insufficient cerebrospinal fluid production, varied cerebral venous blood flow; West, 2004; Luks et al., 2017). While there is substantial research on potential contributors to AMS (reviewed in Imray et al., 2010; Luks et al., 2017), the exact biological pathways and molecular mechanisms behind AMS development remain unclear.

Recent studies support a potential role of inflammation in AMS. There is a general consensus that pro-inflammatory cytokines and other inflammatory markers (most notably C-Reactive protein (CRP), IL-1β, and IL-6) are increased in individuals acutely exposed to hypoxia or high altitude (Hartmann et al., 2000; Song et al., 2016; Lundeberg et al., 2018; Wang et al., 2018; Malacrida et al., 2019; Kammerer et al., 2020). In some cases, inflammatory mediator expression appears to differ across individuals who develop AMS and those who do not. For example, individuals who develop AMS have been reported to show decreased plasma levels of IL-10 and increased IL-1β, IL-6, and TNF-α compared to non-AMS controls (Liu et al., 2017; Wang et al., 2018). Furthermore, the association between plasma IL-6 and AMS score has been identified by multiple independent groups (Boos et al., 2016; Wang et al., 2018).

While these studies indicate that some circulating pro-inflammatory markers may be associated with AMS development, it is not yet clear what specific role they may play. It is possible that hypoxia initiates the release of inflammatory and angiogenic mediators which disrupt the blood-brain barrier and promote vasogenic edema. A complementary hypothesis is that protection from AMS may be driven by a robust anti-inflammatory response which protects against potential increased blood-brain barrier permeability caused by acute systemic inflammation (Varatharaj and Galea, 2017). This is supported by data from Julian et al. (2011), who found higher levels of anti-inflammatory marker (IL-1RA, HSP70, and adrenomedullin) expression in AMS-resistant compared to AMS-susceptible individuals (Julian et al., 2011).

Finally, both steroids and nonsteroidal anti-inflammatory drugs (NSAIDS) equally reduce AMS incidence despite their different modes of action (Dumont et al., 2000; Gertsch et al., 2012; Zheng et al., 2014). This suggests that both COX-2 mediated inflammation as well as analgesic mechanisms that mediate nociception contribute to AMS symptomology (Hartmann et al., 2000; Song et al., 2016; Kanaan et al., 2017). Indeed, inflammatory mediators released during tissue injury can activate nociceptors and can contribute to pain hypersensitivity (Kidd and Urban, 2001). However, Lundeberg et al. (2018) note no change in AMS symptoms in individuals receiving Ibuprofen at normal recommended doses of 400mg three times a day (Lundeberg et al., 2018), perhaps, due to the lower dosage compared to other studies which did observe a reduction in AMS symptoms with a higher Ibuprofen dose (600 mg three times a day; Gertsch et al., 2010, 2012; Lipman et al., 2012).

#### High Altitude Cerebral Edema

HACE is a severe and potentially fatal complication that can occur in individuals who travel above 2000 m. HACE is accompanied by symptoms including headache, ataxia, declines in cognitive function, and can lead to seizures (Hackett, 1999). As with AMS, HACE is most common with rapid ascent. HACE is frequently preceded by some AMS symptoms, and both illnesses are influenced by cerebral hemodynamics. As a result, HACE is sometimes considered a more severe form of AMS, however, additional distinct pathophysiological mechanisms contribute to HACE development (Hackett and Roach, 2004; Brugniaux et al., 2007; Li et al., 2018). HACE can develop spontaneously at very high altitudes in acclimatized individuals (Clarke, 1988). HACE appears to occur as a result of hypoxia-mediated cerebral vasodilation and a subsequent impairment of the autoregulation of cerebral blood flow, loss of blood-brain barrier integrity, and a rise in intracranial pressure (Hackett, 2000). Ultimately, cytotoxic and vasogenic edema leads to microvascular disruption and microbleeds (Hackett et al., 2019).

While some aspects of HACE pathophysiology remain uncertain, the underlying mechanisms producing HACE are likely similar to those of AMS and inflammation may play a role in its development (Bailey et al., 2009; Song et al., 2016; Zhou et al., 2017). In both mouse and rat models, pro-inflammatory cytokines are significantly increased in the brain cortex after hypoxia exposure. Furthermore, when pre-treated with bacterial LPS to produce a systemic inflammatory response, subsequent hypoxia exposure results in cerebral edema (Song et al., 2016; Zhou et al., 2017). It is proposed that pre-existing inflammation increases aquaporin 4 (AQP4) activity in astrocytes *via* toll-like receptor 4 (TLR4), mitogen-activated protein kinase (MAPK), and NF-κB signaling, thereby increasing astrocyte permeability. Together, these data indicate that when challenged with a combination of hypoxia and inflammation, the combination of increased pro-inflammatory cytokines and increased blood vessel permeability produces vasogenic edema (Song et al., 2016; Zhou et al., 2017).

Although this data provide intriguing support for a role of hypoxia-induced inflammation and HIF-NF-kB crosstalk in HACE onset, it remains unclear if inflammation is a proximal cause of AMS or HACE. Nonetheless, inflammation likely plays a contributing role in susceptibility and progression of these illnesses. Further work is required to reach a unified explanation for HACE development.

#### High-Altitude Pulmonary Hypertension and High-Altitude Pulmonary Edema

HAPH occurs due to general hypoxic pulmonary vasoconstriction (HPV). Under typical conditions, local HPV aids in redistributing blood flow away from lung regions with poor ventilation and improves pulmonary gas exchange. However, at high altitude, global reductions in alveolar oxygen pressure can lead to HPV throughout the lung and increase mean pulmonary artery pressures (Swenson, 2013). HAPH is estimated to impact up to 10% of high-altitude residents (León-Velarde et al., 2005). The clinical presentation of HAPH includes dyspnea, general fatigue, exercise intolerance, and in severe cases, chest pain and mental alterations, and ultimately cor pulmonale.

While acute HAPH is primarily driven by increased vasomotor tone, chronic hypoxic stress and persistent pulmonary hypertension can produce vascular remodeling and exacerbate pulmonary artery pressures (Groves et al., 1987; Wilkins et al., 2015). The hallmark of vascular remodeling in chronic hypoxia is increased vessel muscularization (Arias-Stella and Saldana, 1963; Hislop and Reid, 1976). It is predicted that inflammation plays an important role in this remodeling process. Chronic hypoxia produces a proinflammatory microenvironment in pulmonary artery walls (Burke et al., 2009). Resident fibroblasts, immune cells, and progenitor cells in the vascular adventitia respond to this local cellular stress by releasing additional inflammatory mediators and growth factors which impact vascular wall cell phenotypes and contribute to increased muscularization (Stenmark et al., 2013). This inflammatory microenvironment also promotes recruitment, retention, and differentiation of additional inflammatory cells (Burke et al., 2009).

HAPE occurs most commonly during rapid ascent. This is due to exaggerated HPV which causes acute pulmonary hypertension, increased capillary permeability, and alveolar fluid buildup (Talbot et al., 2005; Dunham-Snary et al., 2017; Brito et al., 2020; Swenson, 2020; Sydykov et al., 2021). HAPE onset is primarily due to this non-inflammatory hydrostatic pulmonary edema (Swenson et al., 2002). However, inflammation may play a downstream role in HAPE pathology. Several studies report increased inflammatory mediators and chemokines in bronchoalveolar fluid in later stages of the illness including leukotriene B_4_ and complement fragments (C5a; Schoene et al., 1986, 1988), plasma endothelin-1 (ET-1; Droma et al., 1996), lactate dehydrogenase, IL-1β, IL-6, IL-8, TNF-α, and IL-1RA (Kubo et al., 1996, 1998). Several of these studies also report increased leukocyte counts in bronchoalveolar lavage fluid. This late-stage inflammation could exacerbate pulmonary vascular leakage by causing endothelial damage and increasing capillary permeability (Mishra et al., 2016).

Some studies have also suggested that inflammation contributes to HAPE susceptibility. Individuals with a history of HAPE are more susceptible to developing HAPE again upon re-entry to high altitude (Lakshminarayan and Pierson, 1975; Bärtsch, 1999; Gallagher and Hackett, 2004). This increased susceptibility is attributed to higher baseline inflammation (Mishra et al., 2016). Furthermore, one report suggests that HAPE-susceptible individuals also demonstrate reduced lung function compared to HAPE-resistant individuals (Gupta et al., 2017), which would limit the adaptive compensatory ventilatory response to hypoxia. The reduced lung function in these individuals is further correlated with increased plasma CRP (Shaaban et al., 2006; Hancox et al., 2007). This association between chronic inflammation and poor lung function is hypothesized to contribute to HAPE-susceptibility, although it does not explain the direct cause of onset. Additionally, pre-existing pulmonary vascular remodeling, driven partially by inflammation, may exacerbate pulmonary pressures and blood flow distribution and thereby increase HAPE susceptibility (Wilkins et al., 2015).

### Inflammation and Erythropoiesis

Erythropoiesis is tightly regulated to maintain homeostatic balance between red blood cell production and degradation. This balance is crucial for optimal oxygen delivery to tissues. An inadequate number of erythrocytes lead to tissue hypoxia, while high erythrocyte concentrations can increase blood viscosity, impair blood flow, and increase risk of thrombosis (Keohane et al., 2013). This is particularly relevant in Chronic Mountain Sickness (CMS), a clinical syndrome commonly presented in high-altitude natives and life-long residents, which frequently coincides with excessive erythrocytosis (Hb ≥ 19 g/dL for women, Hb ≥ 21 g/dL for men) in some high altitude groups (León-Velarde et al., 2005; Oberholzer et al., 2020). The HIF pathway plays a key role in regulating erythropoiesis. HIF activation results in increased transcription of the *EPO* gene in the kidney and liver, and EPO serum concentration can increase up to several 100-fold in response (Imagawa et al., 1990; Haase, 2013). Additionally, HIF activity plays a role in regulating iron uptake from the gut and mobilizing erythroid progenitor cells in the bone marrow (Nemeth, 2008).

Chronic inflammation typically leads to anemia through several mechanisms: prioritizing myeloid cell production, sequestering iron, and increasing erythroid turnover rate. Inflammation impacts crucial sites of erythrocyte production to redistribute resources toward myeloid cell production and therefore reduces lymphoid and erythroid output. For example, expression of pro-inflammatory IL-1β, interferon gamma (IFNγ), and IL-6 skew multipotential hematopoietic progenitors toward myeloid cell development (Tie et al., 2019). This is an adaptive response since myeloid cells are needed to fight infection; however, this comes at the cost of bone marrow erythropoiesis. Additionally, while iron is an essential component of hemoglobin synthesis, it is also an essential micronutrient to pathogens. During inflammation, increased IL-6 induces hepcidin production, interrupting iron absorption from the gut and blocking iron release from macrophages, leading to hypoferremia (Nemeth et al., 2004; Nemeth, 2008; Zaritsky et al., 2009; Weiss et al., 2019). By decreasing iron availability, the immune system limits pathogen proliferation; however, a consequence of iron sequestration is that this also limits iron availability for red blood cell production.

When steady state erythropoiesis becomes insufficient, or red blood cells are broken down at high rates, a compensatory extramedullary mechanism called stress erythropoiesis is initiated to prevent lethal anemia (Paulson et al., 2011). Stress erythrocytosis produces a burst of new erythrocytes to maintain red cell concentrations until bone marrow erythropoiesis recovers. The mechanism of stress erythropoiesis has been studied extensively in mice, where it occurs in the spleen and liver in response to anemia, hypoxia, or sterile inflammation. Studies in mice revealed that sterile inflammation produced by phenylhydrazine injections lead to decreased erythroid progenitor cells in the bone marrow and increased erythroid progenitor cells in the spleen. It was discovered that bone marrow erythroid progenitor cells and splenic erythroid progenitor cells respond to different factors, indicating that splenic progenitors are distinct from bone marrow progenitors. This splenic stress erythropoiesis response depends on TLR signaling molecules Myd88 and TRIF (Jackson et al., 2010). Furthermore, pro-inflammatory cytokines TNF-α and IL-1β promote the expansion of splenic erythroid progenitors (Liao et al., 2018; Bennett et al., 2019). It is currently unknown if similar mechanisms are responsible for stress erythropoiesis in humans (Kim et al., 2015; Mairbäurl, 2018). Future studies may investigate biomarkers of stress erythropoiesis signaling to determine if this protective mechanism is active during high-altitude exposure or other forms of hypoxic stress.

### Impacts of Chronic Hypoxia on Immune Function and Inflammatory Signaling

Immune cells are exposed to hypoxia when they are recruited to sites of inflammation. In physiological immunological niches (bone marrow, placenta, gastrointestinal tract mucosal surfaces, and lymph nodes), the maintenance of sustained and moderate physiological hypoxia is an adaptive mechanism to regulate metabolic pathways and immune homeostasis. However, in pathological immunological niches (tumors and chronically inflamed and ischemic tissue), severe and unregulated hypoxia can lead to maladaptive inflammation and disease development (Taylor and Colgan, 2017). In either case, the immune cell response to hypoxia hinges upon the ability to mount a transcriptional response. Therefore, HIF activity is essential to immune cell survival and function.

Several studies demonstrate the significance of HIF signaling in immune function. HIF-1α deletion in myeloid cells (granulocytes and monocytes/macrophages) impairs their mobility, aggregation, antibacterial activity, and survival (Cramer et al., 2003; Walmsley et al., 2005). On an organismal level, HIF-1α deletion in macrophages can reduce mortality in LPS-induced sepsis in mice (Peyssonnaux et al., 2007). HIF-2α can also directly regulate pro-inflammatory cytokine expression in myeloid cells (Imtiyaz et al., 2010). In addition to HIF’s essential role in immune cell function, NF-kB is also critical to cell survival in hypoxia. Walmsley et al. (2005) demonstrated that the transcription of the NF-κB p65 subunit is regulated by hypoxia. Neutrophils cultured in hypoxia and treated with NF-κB inhibitors had reduced survival rates, suggesting that activation of the NF-κB pathway promotes neutrophil survival in hypoxia (Walmsley et al., 2005).

Despite this important interplay between hypoxia and inflammation in immune function, we know little about how inflammatory signaling and immune function adapt to chronic sustained or chronic intermittent hypoxia. Studies in animal models demonstrate potential blunting or sensitization of inflammatory responses to infection as a result of chronic hypoxia exposure. A study in salmon exposed to chronic hypoxia for 58 days found blunted expression of pro-inflammatory genes in macrophages and the head kidney in response to a viral inflammatory stimulus (Kvamme et al., 2013). Alternatively, in a mouse model, 36 days of hypoxia exposure lead to an enhanced immunological response to LPS illustrated by higher antibody titers and higher TNF-α expression, indicating that hypoxia may stimulate innate and adaptive immune responses (Baze et al., 2011).

Among studies in humans, chronic hypoxia also appears to modulate immune function and inflammatory signaling. A study of women exposed to high altitude (5,050 m) for 21 days showed increased white blood cells, reductions in CD3+ and CD4+ T-cells, an increase in natural killer cells, and a decrease in IFNγ expression by circulating T-cells (Facco et al., 2005). Another recent study in humans exposed to high altitude (3,232 m) for up to 11 months shows that cytokine expression in response to inflammatory stimuli were also higher than sea-level values (Feuerecker et al., 2019). The possibility that chronic hypoxia exposure in humans leads to immune sensitization and hypersensitivity to inflammatory stimuli warrants future study. Since several critical and chronic illnesses are associated with hypoxemia (e.g., sepsis, acute respiratory distress syndrome, chronic obstructive pulmonary disease, and sleep apnea), it is possible that inflammatory dysregulation in these conditions may be caused in part by this concurrent hypoxic stress.

## Lessons From High-Altitude Acclimatized and Adapted Groups

High altitude environments are physiologically stressful due to low atmospheric pressure and oxygen limitation. Despite this, humans have survived in these environments for thousands of years and different high-altitude native populations exhibit distinct physiological adaptations that may be associated with genetic variants. In this section, we discuss what can be learned from high-altitude acclimatized and adapted populations about how inflammatory pathways respond to chronic hypoxia.

### Inflammatory Pathway Genes Under Selection in High-Altitude Groups

Several studies have identified genes under evolutionary selection in high-altitude native populations. HIF pathway genes, particularly *EGLN1* and *EPAS1*, are consistently highlighted across studies. Additionally, several genes associated with inflammation also show signals of selection but have received less attention (Foll et al., 2014). The inflammatory pathway genes *IL6*, *IL1A*, *IL1B*, *NOS1*, *NOS2*, and *TNF* show signals of evolutionary selection in both Andean and Tibetan high-altitude native populations (Beall, 2007; Bigham and Lee, 2014; Moore, 2017). *NOS1* and *NOS3* have also been shown to be under positive selection in the Sherpa population (Droma et al., 2006; Zhang et al., 2017). Additional inflammation-related genes have been reported to be under selection in individual groups, including *HLA-DQB1*, *PPARA*, and *TGFBR3* in Tibetans (Yang et al., 2017), *PPARA* in Sherpas (Horscroft et al., 2017), *BRINP3*, *DUOX2*, and *CLC* in Andeans (Crawford et al., 2017; Jacovas et al., 2018), and *AIMP1* in Ethiopians (Scheinfeldt et al., 2012). Table 1 summarizes inflammation-related genes found to be under evolutionary selection in high altitude human populations.

**Table 1:** Inflammation-related genes identified as top candidates showing signals of evolutionary selection in high-altitude human populations.

Gene name	Protein encoded	Population	Function	Reference
*IL6*	Interleukin-6	Andean, Tibetan	Pro-inflammatory cytokine Anti-inflammatory myokine	Bigham and Lee, 2014 Foll et al., 2014
*TNF*	Tumor necrosis factor, TNF-a	Andean, Tibetan	Pro-inflammatory cytokine	Bigham and Lee, 2014
*IL1A*	Interleukin 1 alpha	Andean, Tibetan	Pro-inflammatory cytokine	Bigham and Lee, 2014
*IL1B*	Interleukin 1 beta	Andean, Tibetan	Pro-inflammatory cytokine	Bigham and Lee, 2014
*HMOX2*	Heme oxygenase 2	Tibetan	Heme protein catabolism	Yang et al., 2016 Peng et al., 2011
*NOS1*	Nitric oxide synthase 1 (neuronal)	Andean, Tibetan, Sherpa, Ethiopian	Nitric oxide production	Bigham and Lee, 2014 Horscroft et al., 2017
*NOS2*	Nitric oxide synthase 2	Andean, Tibetan	Nitric oxide production	Crawford et al., 2017 Bigham and Lee, 2014 Bigham et al., 2010
*NOS3*	Nitric oxide synthase 3 (endothelial)	Sherpa	Nitric oxide production	Droma et al., 2006
*BRINP3*	BMP/Retinoic acid inducible neural specific 3	Andean	Associated with vascular inflammation	Crawford et al., 2017
*CLC*	Galectin-10	Andean	Immune response regulation	Jacovas et al., 2018
*HLA-DQB1*	HLA class II histocompatibility antigen, DQ beta 1 chain	Tibetan	Immune response regulation Detection of foreign proteins	Yang et al., 2017
*AIMP1*	Aminoacyl tRNA synthase complex-interacting multifunctional protein 1	Ethiopian	Inflammatory cytokine activity Involved in angiogenesis, inflammation, wound healing, and glucose homeostasis	Scheinfeldt et al., 2012
*PPARA*	Peroxisome proliferator-activated receptor alpha	Tibetan, Sherpa	Regulation of inflammation and immune response	Simonson et al., 2010 Horscroft et al., 2017
*TGFBR3*	Betaglycan, Transforming growth factor beta receptor type 3	Tibetan	Involved in inflammatory cytokine (TGF-beta) signaling	Peng et al., 2011

*IL6* is particularly interesting given that its expression has been associated with AMS development. IL-6 is also noted to increase hematopoietic stem cell proliferation under hypoxic conditions, and its effects on red cell production are synergistic with other pro-inflammatory cytokines, including IL-1α and TNF-α (Faquin et al., 1992). Together, this data suggest that IL-6 plays a role in adaptation to chronic hypoxia. It is currently unknown what the precise adaptive *IL6* variants are, but it is possible that blunting IL-6 expression in response to chronic hypoxia or chronic intermittent hypoxia may provide an advantage by preventing hypoxia-induced chronic low-grade systemic inflammation. Another important inflammatory gene under selection in both Tibetan and Andean groups is *TNF* (Moore, 2017), a multifunctional pro-inflammatory cytokine. TNF-α levels have also been found to be elevated in individuals traveling acutely to high altitude (Lundeberg et al., 2018). Like *IL6*, the adaptive *TNF* variant is unknown but modulation of *TNF* expression in response to chronic hypoxia may be important for preventing chronic inflammation at high altitude.

In addition to these genetic changes, epigenetic mechanisms also play a critical role in regulating expression of inflammatory genes and likely influence adaptation of inflammatory pathways to chronic hypoxia (Bayarsaihan, 2011). Epigenetic changes alter gene expression without altering the underlying genetic code. These changes include DNA methylation, histone modifications, and microRNA expression. These epigenetic changes play key roles in environmental adaptation during development and exposure in adulthood (Fernández et al., 2014). There are several HIF-dependent and independent mechanisms by which hypoxia significantly impacts epigenetic modifications (Kim and Park, 2020). For example, HIF induces expression of several histone methyltransferases and demethylases (Batie et al., 2019). Additionally, hydroxylation of histone methyl transferases as well as activity of histone demethylases, are oxygen dependent. DNA methylation patterns are also altered by hypoxia, in part due to the impact on ten-eleven translocation (TET) enzyme expression and activity (Thienpont et al., 2016). The HIF consensus binding site also contains a CpG dinucleotide, indicating that expression of HIF-pathway genes may be dependent on DNA methylation (Wenger et al., 2005; Chen et al., 2020; D’Anna et al., 2020). Given that immune cell phenotypes, including inflammatory macrophage phenotypes, can be regulated by epigenetic modifications, hypoxia has the potential to significantly alter immune cell function *via* its impact on epigenetics (Davis and Gallagher, 2019). These changes may occur within or across generations of exposure. Future work should investigate if high-altitude acclimatized or adapted groups develop particular patterns of DNA methylation or histone modification, which protect against chronic hypoxia-induced inflammation. Over generations, mutations at loci containing CpG sites could also assist in blunting inflammatory gene expression in the face of chronic hypoxic stress.

### Nitric Oxide in Native and Acclimatized High-Altitude Groups

Nitric oxide (NO) is a natural vasodilator that plays a crucial role in regulating vasodilation in vascular smooth muscle. Exhaled NO is also used as marker of airway inflammation (Kharitonov and Barnes, 2001; Birrell et al., 2006). The vasodilatory function of NO protects against pulmonary hypertension at high altitude (Feelisch, 2018). Individuals exposed acutely to high altitude tend to show reduced gas phase NO, which typically returns to baseline levels after a couple days then exceeds baseline levels by 5 days. However, gas phase NO has been demonstrated to remain low in HAPE-sensitive individuals, perhaps, indicating that deficits in pulmonary epithelial NO synthesis contribute to exaggerated pulmonary vasoconstriction and edema (Duplain et al., 2000). Tibetan and Bolivian high-altitude adapted populations also show elevated exhaled NO compared to sea-level residents (Beall et al., 2001, 2012; Erzurum et al., 2007). This phenotype correlates with higher pulmonary blood flow and protection from HAPH. However, He et al. (2017) demonstrate that enhanced NO production may not be unique to high-altitude native populations since individuals of Han Chinese ancestry living at high altitude (3,660–3,700 m) demonstrated an even higher average NO metabolite production than Tibetans at the same altitude (He et al., 2017). Another study reports that the native Sherpa population has a lower level of circulating NO in serum and no differences in circulating NO metabolites in comparison to low-altitude samples (Droma et al., 2006; Horscroft et al., 2017). Interestingly, this finding contrasts the high exhaled NO among Tibetans. This discrepancy in NO substrate bioavailability in Tibetans vs. Sherpas also suggests that these two high-altitude populations may have some distinct adaptation in the nitric oxide pathway (Zhang et al., 2017).

Elevated NO may also be an important adaptation of the immune system to chronic hypoxia-induced inflammation. Under normal physiological conditions, NO plays an anti-inflammatory role and inhibits platelet aggregation and rolling, adherence, and transmigration of leukocytes (Grisham et al., 1998; Coleman, 2001). During inflammatory reactions, the production of the inducible form of nitric oxide synthase in many immune cells, including monocytes, macrophages, and neutrophils leads to very large increases in NO by up to 1,000-fold (Cook and Cattell, 1996; Sharma et al., 2007). NO can then become oxidized to reactive nitrogen oxide species which nitrosate thiol groups in glutathione. This inhibits the activity of many proteins including mitochondrial enzymes and transcription factors. As a result, elevated NO production can impair pathogen function (Sethi and Dikshit, 2000; Bogdan, 2001; Coleman, 2001). At high concentrations, NO also stabilizes HIF by inhibiting HIF PHDs (Taylor and Moncada, 2010). Therefore, NO production during periods of inflammation permit increased expression of HIF-pathway genes as a protective mechanism since tissue inflammation often leads to cellular hypoxia. However, Lüneburg et al. (2016) demonstrated in a rat model that while there is an increase in endothelial nitric oxide synthase (eNOS; NOS3) in chronic hypoxia and chronic intermittent hypoxia conditions, NO bioavailability may be impaired due to increases in asymmetric dimethylarginine (ADMA), a competitive nitric oxide synthase inhibitor that regulates NO production, and hypoxia-induced increase in oxidative stress (Lüneburg et al., 2016). ADMA displaces L-arginine, a NOS substrate, and therefore an increase in ADMA concentrations may result in lower NO substrate bioavailability (Brito et al., 2007). Oxidative stress may lead to superoxide radical production, which may mediate NO degradation and attenuate NO bioavailability (Siques et al., 2014).

Since both *NOS1* (nitric oxide synthase 1, neuronal) and *NOS2* (nitric oxide synthase 2, inducible) are under selection in multiple high-altitude groups, it is clear that adjusting NO production is a key adaptive phenotype for life at high altitude. While the precise adaptive mechanism is unknown, it likely involves protection against pulmonary hypertension and/or modulation of hypoxia-induced inflammation.

### Carbon Monoxide in Adapted High-Altitude Groups

Carbon monoxide (CO) is emerging as a potential therapeutic target for inflammatory modulation due to its anti-inflammatory, anti-apoptotic, and anti-proliferative effects (Knauert et al., 2013; Ryter and Choi, 2016). CO is also used as a clinical marker for inflammation and red blood cell turnover since endogenous CO is only produced through the heme oxygenase (HO) pathway (Maines, 1997). HO is responsible for catabolizing free heme proteins into Fe^2+^, CO, and biliverdin. These end products provide cytoprotective benefits and protect cells from programmed cell death in response to pro-inflammatory agonists (Gozzelino et al., 2010). The HO-CO pathway may be a key regulator of hypoxia-induced inflammation since the gene encoding heme oxygenase-1 (*HMOX1*) is upregulated in response to HIF-pathway activation and HO-1 can inhibit NF-κB (Seldon et al., 2007).

High altitude exposure has been shown to increased exhaled CO levels, and carboxyhemoglobin levels are positively associated with hematocrit in Andean high-altitude residents (Tift et al., 2020). This is suspected to be caused by increased red blood cell turnover, a persistent stress erythropoiesis response, or a unique HO/CO pathway mechanism. Interestingly, the gene encoding the constitutively expressed heme oxygenase-2 (*HMOX2*) is under evolutionary selection in Tibetan high-altitude native groups (Yang et al., 2016). The adaptive *HMOX2* variants in Tibetans are predicted to play a critical role in regulating red blood cell turnover and potentially contribute to the low hemoglobin concentrations in this group by increasing heme oxygenase activity. Due to the key role that the HO-CO pathway plays in linking the molecular responses to hypoxia and inflammation, as well as evidence that HO is a top candidate for evolutionary selection in high-altitude populations, future research should continue to investigate potential therapeutic uses of exogenous CO and HO pathway activation for modulating inflammatory responses, especially in hypoxia-promoted pathologies.

## Conclusion

HIF-NF-κB crosstalk plays an essential role in the transcriptional response to both hypoxia and inflammation. However, additional research is necessary to understand the physiological implications of these pathway interactions at an organismal level. Several studies have demonstrated that select inflammatory mediators are upregulated at high altitude, or in the presence of acute hypoxia, even in the absence of infection. Since these studies have focused on candidate inflammatory markers, large scale transcriptomic or proteomic studies would provide a better understanding of how inflammatory networks are shifted during chronic hypoxia. Further, our understanding of the time domains of hypoxia-induced inflammation and the impact on immune function may also provide insight into pathology of high-altitude illnesses and highlight the importance of hypoxia-induced inflammatory sensitization in critical and chronic illnesses including chronic obstructive pulmonary disorder, sepsis, and COVID-19. This work may identify novel therapeutic targets for mitigating excessive inflammation in patients with concomitant hypoxemia and systemic inflammation.

## Author Contributions

KaP drafted versions of the manuscript with input and revisions from KeP and EH. All authors contributed to the article and approved the submitted version.

### Conflict of Interest

The authors declare that the research was conducted in the absence of any commercial or financial relationships that could be construed as a potential conflict of interest.
